# The amniotic fluid proteome changes with term labor and informs biomarker discovery in maternal plasma

**DOI:** 10.1038/s41598-023-28157-3

**Published:** 2023-02-23

**Authors:** Gaurav Bhatti, Roberto Romero, Nardhy Gomez-Lopez, Tinnakorn Chaiworapongsa, Nandor Gabor Than, Kevin R. Theis, Jose Galaz, Francesca Gotsch, Roger Pique-Regi, Stanley M. Berry, Mahendra Kavdia, Adi L. Tarca

**Affiliations:** 1 Pregnancy Research Branch, Eunice Kennedy Shriver National Institute of Child Health and Human Development, National Institutes of Health, US Department of Health and Human Services, NICHD/NIH/DHHS, Detroit, MI USA; 2 Department of Obstetrics and Gynecology, Wayne State University School of Medicine, Detroit, MI USA; 3 Department of Obstetrics and Gynecology, University of Michigan, Ann Arbor, MI USA; 4 Department of Epidemiology and Biostatistics, Michigan State University, East Lansing, MI USA; 5 Center for Molecular Medicine and Genetics, Wayne State University, Detroit, MI USA; 6 Detroit Medical Center, Detroit, MI USA; 7 Department of Biochemistry, Microbiology and Immunology, Wayne State University School of Medicine, Detroit, MI USA; 8 Systems Biology of Reproduction Research Group, Institute of Enzymology, Research Centre for Natural Sciences, Budapest, Hungary; 9 Maternity Private Clinic, Budapest, Hungary; 10 Department of Obstetrics and Gynecology, Semmelweis University, Budapest, Hungary; 11 Department of Biomedical Engineering, Wayne State University College of Engineering, Detroit, MI USA; 12 Department of Computer Science, Wayne State University College of Engineering, Detroit, MI USA

**Keywords:** Proteome informatics, Diagnostic markers, Diagnostic markers, Intrauterine growth, Preterm birth, Proteomics

## Abstract

The intra-uterine components of labor, namely, myometrial contractility, cervical ripening, and decidua/membrane activation, have been extensively characterized and involve a local pro-inflammatory milieu of cellular and soluble immune mediators. Targeted profiling has demonstrated that such processes extend to the intra-amniotic space, yet unbiased analyses of the proteome of human amniotic fluid during labor are lacking. Herein, we utilized an aptamer-based platform to characterize 1,310 amniotic fluid proteins and found that the proteome undergoes substantial changes with term labor (251 proteins with differential abundance, q < 0.1, and fold change > 1.25). Proteins with increased abundance in labor are enriched for immune and inflammatory processes, consistent with prior reports of labor-associated changes in the intra-uterine space. By integrating the amniotic fluid proteome with previously generated placental-derived single-cell RNA-seq data, we demonstrated the labor-driven upregulation of signatures corresponding to stromal-3 and decidual cells. We also determined that changes in amniotic fluid protein abundance are reflected in the maternal plasma proteome. Collectively, these findings provide novel insights into the amniotic fluid proteome in term labor and support its potential use as a source of biomarkers to distinguish between true and false labor by using maternal blood samples.

## Introduction

Labor is a well-orchestrated process that includes physiological, biochemical, endocrinological, and immunological pathways in the mother and the fetus, culminating in a successful delivery. This complex process involves intra-uterine and extra-uterine components, with the former consisting of increased myometrial contractility, cervical ripening, and decidual/membrane activation^1–4^. A strong body of evidence indicates that the intra-uterine components include a local pro-inflammatory milieu characterized by increased concentration of soluble and number of cellular immune mediators in the myometrium^5–16^, cervix^5,7–9,17–25^, decidua^7,8,26–35^, and chorioamniotic membranes^7,8,27,28,30,36–40^. Such an intra-uterine inflammatory response is also reflected in the intra-amniotic space. Indeed, prior targeted studies have shown that the concentrations of several cytokines, including Interleukin-1β (IL-1β)^41^ and Interleukin-6 (IL-6)^42^, are elevated in the amniotic fluid of women with term labor compared to those without labor. Furthermore, cytomic approaches have shown that innate immune cells, such as neutrophils and monocytes/macrophages, are abundant in the amniotic fluid of women at term gestation^43^. In line with this concept, we recently profiled the amniotic fluid proteome from early to late pregnancy and found that immune-specific signatures were enriched in term gestation^44^. Notably, placental- and uterine-derived signatures were modulated in the amniotic fluid proteome^44^, showing that the fetal and maternal components are present in the intra-amniotic space. Taken together, these findings led us to hypothesize that amniotic fluid harbors cellular and soluble mediators at the end of gestation in preparation for the physiologic intra-amniotic inflammatory process of term parturition. Hence, the primary aim of the current study was to explore the human proteome of amniotic fluid during term parturition.

In addition to intra-uterine components, labor also includes extra-uterine components such as processes taking place in the maternal circulation^1–4^. Indeed, the detection of cell-free RNA and DNA derived from the fetus has been an invaluable clinical tool in evaluating fetal development as well as in screening for fetal abnormalities and obstetrical disease^45–59^. Moreover, the use of high-dimensional techniques such as transcriptomics, proteomics, and metabolomics has allowed for the exploration of the complex and dynamic processes that are modulated in the maternal circulation prior to and during labor^60–70^. Recently, we leveraged the maternal blood transcriptome to monitor single-cell signatures derived from placental tissue and demonstrated the increasing abundance of such transcripts with advancing gestation^71^ as well as in women with term^71^ or spontaneous preterm labor and birth^66^. Similarly, labor-specific transcriptomic changes in the maternal circulation can be correlated with those derived from the chorioamniotic membranes, cervix, or myometrium, suggesting that tissue signatures of labor are partly mirrored in this compartment^70^. By intersecting single-cell RNA-seq data of the laboring myometrium with maternal transcriptomic datasets, we also demonstrated that specific cell signatures were modulated throughout gestation and enriched with the process of labor^72^. Together, these prior observations demonstrate that labor-specific changes taking place in the gestational and reproductive tissues can be tracked in the maternal circulation. Yet, whether the processes occurring in the amniotic fluid during term labor are also reflected in the maternal circulation has not been investigated.

Herein, we utilized the aptamer-based SOMAscan proteomics platform^73^ to characterize changes in the concentrations of 1,310 amniotic fluid proteins during the normal process of labor at term. In addition to identifying amniotic fluid proteins dysregulated with labor, we tested in independent patient sets whether the amniotic fluid-derived signature can discriminate between women in labor and those not in labor based on blood protein profiles.

## Results

### Clinical characteristics of the study population

**Figure 1:**
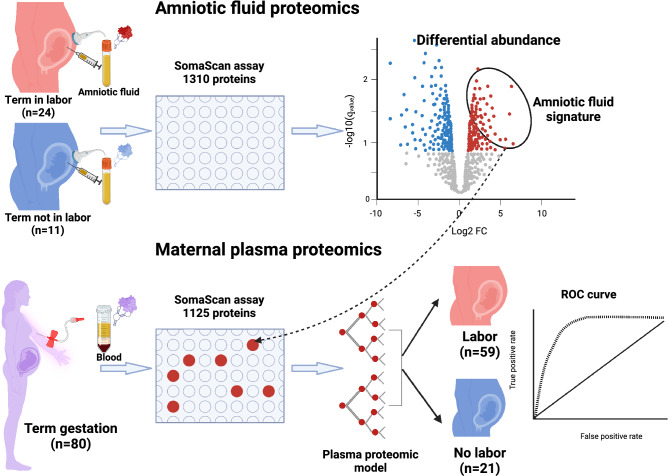
Study design and summary. The study included the determination of 1310 protein analytes in amniotic fluid samples collected at term from patients not in labor (TNL, n = 11) and from those in active labor (TIL, n = 24) (top panel). Maternal plasma concentrations of amniotic fluid proteins that significantly increased in abundance with labor were then used to discriminate labor from no labor groups in an independent set of pregnant women (TNL, n = 21 vs TIL, n = 59) (bottom panel). The figure was created with biorender.com^155^.

This study included amniotic fluid samples collected from women at term in labor (TIL, n = 24) or not in labor (TNL, n = 11) (Fig. 1, top panel). The clinical characteristics of the study participants are shown in Table 1. There were no significant differences in gestational age at amniocentesis between the TIL and TNL patients (median gestational age at amniocentesis: TIL 39 weeks vs. TNL 38 weeks, p = 0.25). There were no significant differences in maternal age, nulliparity, or birth weight between the two groups (Table 1). All women delivered a healthy neonate (Apgar score at 5 min > 8 for all cases) without major pregnancy or neonatal complications.

**Table 1:** Demographic characteristics of the amniotic fluid study cohort.

Characteristics	Term no labor (n = 11)	Term in labor (n = 24)	p
Age (years)	27 (22.5–30)	21 (19.8–23.2)	0.138
Nulliparity	4/10 (40%)	14/24 (58.3%)	0.457
Gestational age at amniocentesis (weeks)	38 (37.2–39)	39 (37.9–39.7)	0.251
Cervical dilatation (cm)		3(3–4)	
Birth weight (g)	3130 (3055–3525)	3400 (3037.5–3600)	0.638

Continuous variables were compared with the use of Welch’s t-test and are summarized as the median (interquartile range). Categorical variables are shown as a number (%) and were compared with the use of Fisher’s exact test.

An additional set of plasma proteome data were available from a separate cohort of TIL (n = 59) and TNL (n = 21) patients (Fig. 1, bottom panel)^74^. The clinical characteristics of the study participants are shown in Supplementary Table S1. There was no significant difference in gestational age at sampling between the two groups (median gestational age at blood draw: TIL 39 weeks vs. TNL 38.7 weeks, p = 0.21).

### Validation of the SOMAmer assay to evaluate amniotic fluid proteins

**Figure 2:**
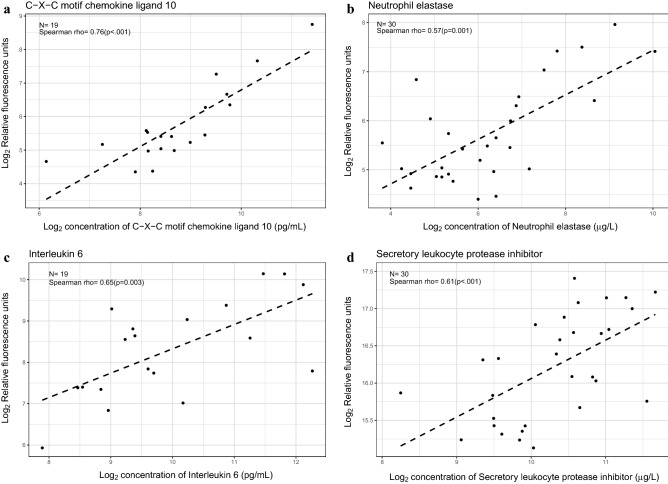
Cross-platform correlation of specific amniotic fluid proteins. Scatter plots of log_2_-transformed relative fluorescence units determined by using the SOMAscan assay (y-axis) and log_2_-transformed ELISA or RIA concentrations (x-axis) for (**a**) C–X–C motif chemokine ligand 10 (CXCL10), (**b**) neutrophil elastase (ELANE), (**c**) interleukin (IL)-6, and (**d**) secretory leukocyte protease inhibitor (SLPI). The number of sample pairs, Spearman's correlation coefficient, and the corresponding p-value are shown per analyte. The scatter plots were generated with the R package, *ggplot2*^159^.

Although the SOMAscan v3 platform has been previously validated by using both targeted and unbiased proteomic platforms across a range of biological specimens, there is limited information about the cross-platform agreement when measuring amniotic fluid proteins^75,76^. Therefore, we utilized enzyme-linked immunoassay (ELISA)-based concentrations of C–X–C motif chemokine ligand 10 (CXCL10), neutrophil elastase (ELANE), interleukin (IL)-6, and secretory leukocyte protease inhibitor (SLPI) that were available for a subset of samples included in this study (Fig. 2). We found a significant positive correlation between the SOMAmer assay and the ELISA for each of these proteins [CXCL10: n = 19, Spearman's ρ = 0.76, p < 0.001; ELANE, n = 30, ρ = 0.57, p = 0.001; IL-6: n = 19, ρ = 0.65, p = 0.003; SLPI: n = 30, ρ = 0.61, p < 0.001], therefore indicating significant cross-platform agreement.

### The amniotic fluid proteome shows changes with labor at term

To visualize the relationship between the amniotic fluid proteome of TIL and TNL patients, principal component (PC) analysis was utilized. Despite some overlap, most amniotic fluid samples showed clear separation according to labor status (Supplementary Fig. S1). Furthermore, the meta-proteomes (PC1 and PC2, 22% and 16% of variance explained, respectively) were significantly associated with labor status (t-test, p < 0.05 for PC1 and for PC2).

Although there was no significant difference in gestational age at amniocentesis between the two study groups (Table 1), we and other investigators have shown that gestational age is a strong modulator of the amniotic fluid proteome and transcriptome, and even a small difference in gestational age at amniocentesis could potentially translate into changes in protein abundance^44,77–79^. However, Pearson correlation analysis showed no evidence of an association between gestational age at amniocentesis and the principal components of our current data [PC1: ρ = 0.01, p = 0.94, PC2: ρ = 0.03, p = 0.89)]. This finding is most likely due to the narrow range of gestational ages wherein amniocentesis was performed in the current study. Thus, we have not adjusted for gestational age at amniocentesis in our subsequent analyses.

**Figure 3:**
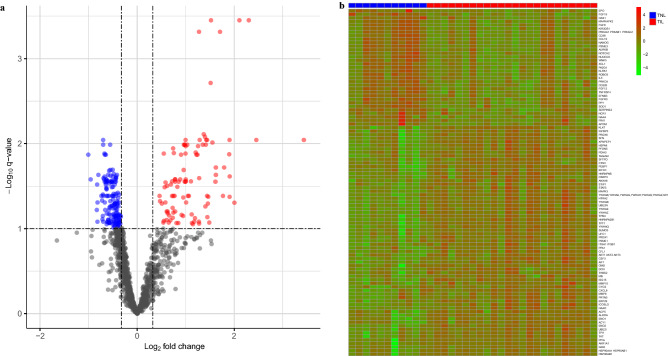
Differential protein abundance in the amniotic fluid with and without labor at term. (**a**) Volcano plot showing log_10_-transformed false discovery rate adjusted *p*-values (q-values) against log_2_-transformed fold changes of the 1310 amniotic fluid proteins. (**b**) Heatmap based on proteins with significantly altered abundance (q<0.05 and fold change ≥ 1.5) between term not in labor (TNL, indicated by blue headers) and term in labor (TIL, indicated by red headers) samples. The R/Bioconductor packages, *EnhancedVolcano*^157^ and *pheatmap*^158^, were used to generate the volcano plot and heatmap, respectively.

A comparison of amniotic fluid protein abundance between the TIL and TNL groups resulted in 251 (19.2%) proteins with significant differences (q < 0.1 and fold change ≥ 1.25) (Supplementary Table S2). Of these, 100 (39.8%) proteins were more abundant, and 151 (60.2%) proteins were less abundant in TIL than in TNL. The summary results of this differential abundance analysis are displayed in Fig. 3, where the volcano plot indicates the magnitude of differences in protein abundance (log_2_ fold changes) against the statistical significance (q-values) of these differences (Fig. 3a). In addition, the heat map shown in Fig. 3b displays the log_2_ abundance in relative fluorescence units of the most significant (q < 0.05 and fold change ≥ 1.5) proteins in a color scale, indicating consistent patterns of protein abundance within each study group. These results illustrate the distinct amniotic fluid proteomic profile that characterizes labor at term.

### Proteins differentially abundant with term labor are enriched for distinct signaling processes

Next, to aid in the interpretation of the proteomic dysregulation associated with term labor, we performed gene set enrichment analysis (GSEA) of the MSigDB C5 gene set collection and identified Gene Ontology (GO) terms (biological processes, molecular functions, and cellular components) associated with labor status. We identified 184 biological processes, 24 molecular functions, and 9 cellular components that were enriched in proteins with higher abundance in the TNL compared to TIL samples (q < 0.25) (Supplementary Table S3). Such biological processes included regulation of CD4 positive alpha–beta T-cell differentiation, lymphocyte activation, regulation of protein phosphorylation, cell–cell signaling, tyrosine phosphorylation of STAT protein, regulation of phospholipase activity, cardiac conduction system development, and transmission of nerve impulse (Supplementary Table S3). For proteins that were more abundant in the TIL group compared to the TNL group, we determined the enrichment of 65 biological processes, 28 molecular functions, and 22 cellular components (Supplementary Table S3). The significantly enriched biological processes included regulation of proteolysis, intracellular transport, macromolecule catabolic process, erythrocyte, and myeloid cell homeostasis (Supplementary Table S3).

**Figure 4:**
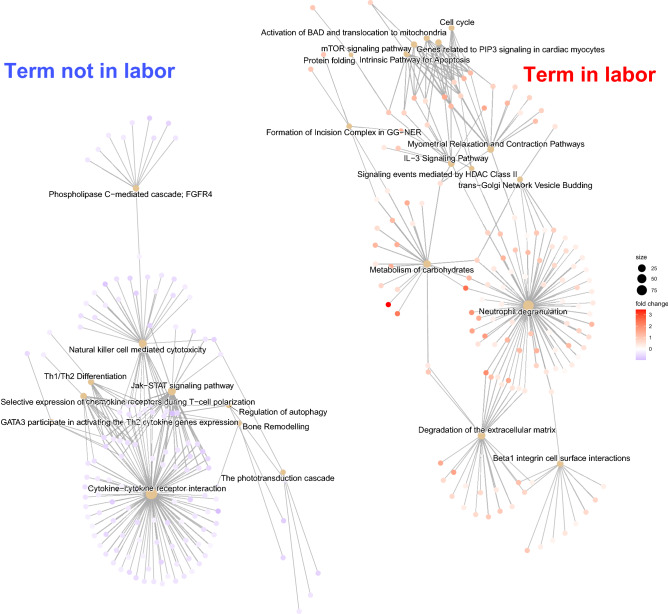
Functional enrichment of biological pathways in the amniotic fluid proteome with and without labor at term. Gene-concept network (cnetplot) of biological pathways that were significantly enriched (q-value < 0.1) before (blue) or after (red) the onset of labor. The size of the nodes, corresponding to enriched terms, represents the number of member genes that contributed to the enrichment (leading edge) of the term. The color of the nodes representing each leading-edge gene indicates the TIL/TNL fold change in the corresponding protein abundance. The relationships between the enriched terms are represented by the shared genes. The R/Bioconductor package, *clusterProfiler*^162^ was used to generate the figure.

We then conducted a GSEA of the MSigDB C2 gene set collection of canonical pathways to gain further insights into the physiology of normal labor as captured by the amniotic fluid proteome. GSEA identified 48 biological pathways that were significantly enriched among proteins more abundant in TNL compared to TIL samples (Supplementary Table S4), which included pathways related to cytokine signaling in T helper cells, e.g., cytokine-cytokine receptor interaction, JAK-STAT signaling, and selective expression of chemokine receptors during T-cell polarization as well as other signaling pathways (Fig. 4). By contrast, the 91 biological pathways enriched among proteins more abundant in TIL samples (Supplementary Table S4) were related to neutrophil degranulation, carbohydrate metabolism, degradation of the extracellular matrix, and myometrial relaxation and contraction pathways.

Taken together, these findings indicate a distinct inflammatory proteomic signature associated with physiological labor at term compared to term no labor.

### Integration of the amniotic fluid proteome with placental single-cell RNA-seq signatures reveals labor-specific enrichment of specific cell types

**Figure 5:**
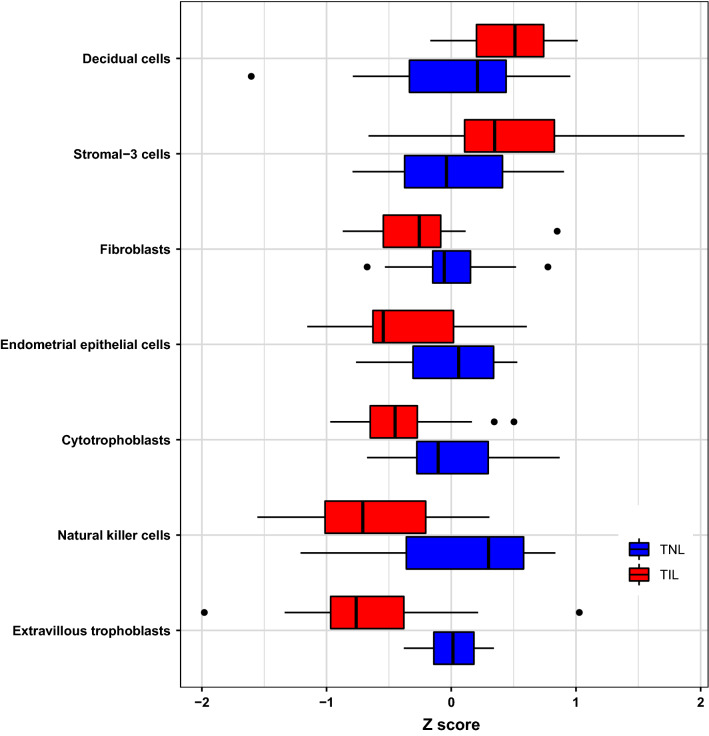
Placental single-cell RNA-seq signatures represented within the amniotic fluid proteome. For each placental cell type signature previously derived by single-cell RNA-seq analysis, the concentration of proteins coded by up to 20 most preferentially expressed genes was transformed into a Z-score and averaged. The Z-scores were compared between the term not in labor (TNL, blue bars) and term in labor (TIL, red bars) groups. The cell types that significantly (q-value < 0.1) changed in expression are shown. The box plots were generated with the R package, *ggplot2*^159^.

Given the crucial role of the placenta in cross-talk between the mother and the fetus during pregnancy and at labor onset, we further sought to interpret the labor-associated changes in the amniotic fluid proteome by intersecting these data with cell type signatures previously identified by using single-cell RNA-seq of placental tissues^66^. The aggregated proteomic signature of decidual cells and stromal-3 cells was significantly increased (q < 0.1) in amniotic fluid samples from TIL patients compared to TNL patients (Fig. 5, Supplementary Table S5). By contrast, there was a significant decrease in the signatures corresponding to extravillous trophoblasts, cytotrophoblasts, natural killer cells, endometrial cells, and fibroblasts with the onset of term labor (Fig. 5, Supplementary Table S5).

### Changes in the amniotic fluid proteome are reflected in the maternal circulation and can distinguish term labor

**Figure 6:**
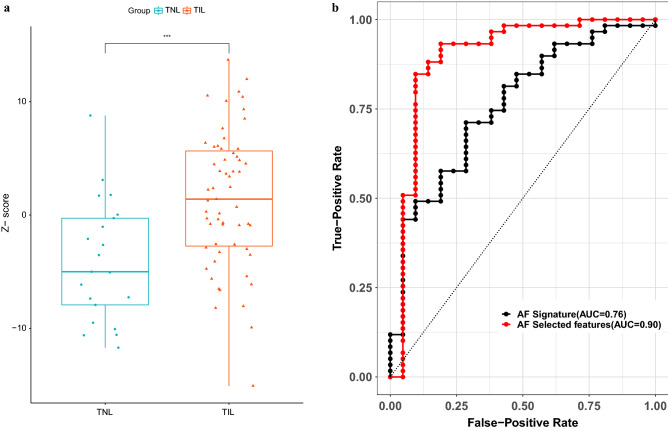
Changes in the amniotic fluid proteome are reflected in the maternal circulation and can distinguish term labor. (**a**) Boxplots showing changes in the amniotic fluid-derived labor signature in the maternal plasma proteomic profiles. The box plots were generated with the R package, *ggpubr*^163^. (**b**) Receiver operating characteristic curves for classifying maternal plasma samples into labor and no labor groups based on the plasma concentrations of proteins identified in the amniotic fluid. The black curve corresponds to the discrimination accuracy of the aggregated proteomic signature of the top 15 amniotic fluid proteins that were most increased in the TIL group compared to the TNL group. The red curve corresponds to the leave-one-out cross-validation (LOOCV)-based predictions of a random forest model trained with 10 proteins selected according to their importance from all amniotic fluid proteins increased in the TIL group compared to the TNL group. The ROC curves were plotted with the R package, *pROC*^164^.

We then sought to determine whether the labor-associated protein changes in amniotic fluid are also reflected in the maternal plasma proteome, given that signatures derived from the tissues surrounding this compartment can be monitored in the maternal circulation^66,70,72^. We first defined an amniotic fluid proteomic signature of term labor by selecting the top 20 proteins that were most increased in the TIL group compared to the TNL group. Among these 20 proteins, the abundance of 15 in the maternal plasma was also available in a dataset generated by SOMAScan v.2 in an independent set of uncomplicated pregnancies^74^. The aggregated proteomic signature (average of Z-scores) was significantly higher in maternal plasma samples collected at term labor compared to gestational age-matched samples collected from women not in labor (Fig. 6a), and the discrimination accuracy was substantial (area under the curve [AUC] = 0.76, 95% confidence interval [CI] 0.64–0.88) (Fig. 6b).

Finally, we extended the pool of protein candidates to all proteins with significantly higher abundance in amniotic fluid during labor (Supplementary Table S2) and allowed a random forest classifier to select and assign different levels of importance to the different candidate proteins, as opposed to the simple average, as described above. Random forest models based on ten proteins selected according to their importance resulted in a leave-one-out cross-validation (LOOCV) AUC of 0.9 (95% CI 0.8–1) (Fig. 6b). Among the considered proteins, those most informative for distinguishing between labor and no labor in maternal plasma samples were ranked high to low: ERP29 (endoplasmic reticulum resident protein 29), SERPINE1 (plasminogen activator inhibitor 1), ICOSLG (ICOS ligand), SFTPD (pulmonary surfactant-associated protein D), HNRNPAB (heterogeneous nuclear ribonucleoprotein A/B), UFC1 (ubiquitin-fold modifier-conjugating enzyme 1), CTSV (cathepsin L2), PSME1 (proteasome activator complex subunit 1), IGFBP1 (insulin-like growth factor-binding protein 1), and PRDX1 (peroxiredoxin-1).

These data demonstrate that labor-associated changes in the amniotic fluid proteome can be monitored in the maternal circulation, providing a potential non-invasive means to distinguish labor.

## Discussion

We utilized an aptamer-based proteomic platform to measure the abundance of 1,310 proteins in amniotic fluid samples collected from women with spontaneous term labor and from those at term without labor. First, we demonstrated that the amniotic fluid proteome undergoes substantial changes with spontaneous term labor that include the altered abundance of 251 proteins. Moreover, such proteins are enriched for immune and inflammatory processes, consistent with labor-associated changes occurring in other compartments. By integrating the amniotic fluid proteome with placenta-derived single-cell RNA-seq data, we demonstrated the labor-driven upregulation of signatures corresponding to decidual and stromal-3 cells. Importantly, we show that changes in amniotic fluid protein abundance are reflected in the maternal plasma proteome, providing the means to distinguish between the patients in labor from those not in labor by using minimally invasive samples. Such data may inform the development of blood tests to distinguish true labor from false labor at term and improve patient management.

Previous investigations have sought to describe the soluble proteome of the amniotic fluid in normal pregnancy by using MS-based approaches^77,80–83^, although such studies did not evaluate changes associated with spontaneous term labor. The proteomics platform utilized herein targets a predetermined number of proteins with specific aptamers, thus allowing for high throughput measurements comparable to immunoassays over a wide dynamic range^75,76,84^. While MS-based approaches identify more proteins, they tend to miss low abundant signaling proteins, e.g., cytokines^85^. The aptamer-based platform has been previously used to describe changes in the amniotic fluid^44^ and maternal plasma proteome^86^ with advancing gestation and pregnancy complications^87–90^; yet, the current study represents its first application to study physiological term labor.

Prior research into amniotic fluid proteins in term labor has primarily utilized targeted approaches to evaluate individual proteins or specific protein sets^41,42,91–123^. Such studies support our current observations by indicating a general increase in inflammatory mediators in amniotic fluid during labor, as evidenced by the elevated concentrations of pro-inflammatory cytokines^41,42,95,99–102,106,111^, chemokines^93,97,99,107,115,121^, arachnoid acid metabolites^124,125^, and extracellular matrix-degrading proteases^108,113,116,126^. Herein, we confirmed the previously reported term labor-associated increase in concentrations of several such inflammatory proteins, including IL-6^42,99–102^, C-X-C motif chemokine ligand 8 (CXCL8)^93,97,99^, colony stimulating factor-3 (CSF3)^96,99^, matrix metallopeptidase-8 (MMP8)^110^, matrix metallopeptidase-9 (MMP9)^108,126^, and heat shock protein family A (Hsp70) member 1A (HSPA1A)^120^. We also reproduced the previously reported decrease in amniotic fluid concentrations of tumor necrosis factor ligand superfamily member 6, soluble form (FASLG)^127^, intercellular adhesion molecule-1 (ICAM1)^122^, and lymphotoxin alpha (LTA)^104^ with labor. Yet, the application of an aptamer-based multiplex proteomics platform has enabled deeper exploration beyond markers of inflammation to describe labor-associated changes in proteins involved in other processes such as intracellular signaling, cell metabolism, intracellular transport, and cell–cell communications.

Herein, pathway analysis of the amniotic fluid protein changes in term labor identified innate immune responses and neutrophil degranulation as being enriched among upregulated proteins. This observation is in line with a previous report of amniotic fluid immunophenotyping, showing that neutrophils are the most abundant cell types in the amniotic fluid at term^43^. Moreover, our previous study comparing the amniotic fluid proteome between samples collected at term (without labor) and those collected during mid-trimester also identified neutrophil-mediated immunity as the most enriched functional term among proteins that increased in concentration at term^44^. Neutrophils produce matrix metalloproteinases that degrade the extracellular matrix, a process crucial for two key events of parturition: rupture of the fetal membranes and cervical ripening^113,128–130^. Indeed, GO and pathway analyses identified terms such as proteolysis, genes encoding enzymes and their regulators involved in the remodeling of the extracellular matrix, and extracellular matrix organization as enriched among proteins with increased abundance during labor.

Another set of functional terms that emerged from the functional profiling of proteins with increased abundance in term labor was related to carbohydrate metabolism. The top three upregulated proteins in labor were glycolytic enzymes: alpha-enolase (ENO1), fructose-bisphosphate aldolase A (ALDOA), and gamma-enolase (ENO2). Parturition requires diverse effector functions of multiple cell types across the maternal and fetal intrauterine tissues, making energy generation and consumption essential for accomplishing parturition without complications. Glucose metabolism meets a major portion of this increased energy demand^131,132^. Other studies of term and preterm pregnancies have also found an increase in the concentrations of proteins involved in carbohydrate metabolism with the spontaneous onset of labor in amniotic fluid and cervicovaginal fluid^133–136^. For example, analysis of paired cervicovaginal fluid samples collected a week before and a week after the onset of spontaneous term labor showed a significant increase in ENO1 with labor^133^. In addition, mass spectral proteomics analysis of amniotic fluid samples collected from women with spontaneous preterm labor has identified glycolysis/gluconeogenesis-associated proteins as differentially abundant in women who delivered preterm with^134^ and without^134,135^ intra-uterine infection or intra-uterine inflammation.

Studies in murine pregnancy have indicated that the fetal lung secretes surfactant components, particularly surfactant protein A (SP-A), into the amniotic fluid, where it can activate macrophages and induce inflammation and parturition^137–139^. However, it was observed that SP-A concentrations were lower in human amniotic fluid samples collected at term in labor compared to those at term without labor^118^. While SP-A was not measured in the current study, a functionally related protein, pulmonary surfactant-associated protein D (SFTPD), was among the most abundant in amniotic fluid samples collected after labor onset. Of note, while a moderate increase in the amniotic fluid concentration of SFTPD in amniotic fluid has been reported throughout the third trimester^140^, we did not previously observe a similar change in the amniotic fluid samples collected at term without labor compared to those collected during mid-pregnancy^44^. Thus, the observed changes in amniotic fluid concentrations of SFTPD could be specific to labor rather than to advanced gestational age. In addition, a genetic study has previously shown a significant association between *SFTPD* gene polymorphism and spontaneous preterm birth among women with recurrent preterm birth^141^.

Moreover, we showed that the meta-protein signature corresponding to decidual cells was increased in amniotic fluid samples collected during labor. Notably, we previously determined that the meta-RNA expression of decidual cells decreases with gestational age in amniotic fluid samples collected before the onset of labor^79^, suggesting that a labor-specific activation of the decidual cells occurs. Indeed, decidual activation is a crucial component of the common pathway of parturition^4,142,143^. Consistently, in addition to the increased meta-protein signature of decidual cells, we also observed that the biological pathway of myometrial contraction and relaxation was enriched among proteins that were more abundant in samples collected after labor. Given that myometrial contractility represents another component of the common pathway of parturition^3,4,142^, these findings further support the reflection of labor-specific changes in the intra-uterine tissues by the amniotic fluid proteome.

Amniocentesis is an invasive procedure that is no longer a part of routine maternal care^144^; thus, the development of diagnostic biomarkers that can be evaluated by using minimally invasive samples, such as maternal blood, is essential. To determine the potential of non-invasive biomarker discovery based on information derived from amniotic fluid, we followed a previously described analytic approach to define and track disease-specific^69^, single cell-specific^79^, and tissue-specific signatures^44,145^ in the maternal circulation and amniotic fluid. First, we defined a labor-specific signature as consisting of amniotic fluid proteins that increased the most in abundance with labor in a Hispanic population. Next, we showed that this meta-protein signature is significantly increased with term labor in the maternal plasma proteome of an independent set of predominantly African-American mothers^74^. The simple average of standardized plasma protein abundance distinguished between the groups, which is encouraging when considering that the proteins were identified in the amniotic fluid analysis. Moreover, when the list of candidate proteins was expanded from the top 15 to all amniotic fluid proteins significantly increased with labor, a plasma proteomic random forest model led to substantially improved accuracy. The set of proteins that most contributed to prediction accuracy included many previously known to be associated with labor in gestational tissues. For example, the expression of the gene coding for SERPINE1 was previously shown to be elevated during labor in the myometrium and placenta^146–149^. Moreover, mass spectrometry-based proteomic analysis of placental membranes collected after spontaneous labor (term or preterm) showed that ERP29 was detected only in term placentas^150^. The concentration of decidual IGFBP1 was shown to increase in vaginal secretions after fetal membrane rupture^151^, and an increased concentration of IGFBP1 in the cervicovaginal fluid has been proposed as a biomarker of PPROM^152–154^. These findings indicate that the amniotic fluid proteomic changes associated with term labor have biological plausibility and are translatable to the maternal circulation and across diverse cohorts of patients.

## Strengths and limitations

The strengths of this study include the unbiased analysis of the proteome (1,310 proteins), although we recognize that this protein set represents a fraction of the human proteome. Another strength is the use of gestational age-matched samples to control for this variable, given that we have demonstrated its influence on the amniotic fluid proteome^44^. Finally, while amniotic fluid proteomic analysis was performed in a Hispanic population, the observed labor-specific changes were translatable to the maternal plasma proteome of a predominantly African-American population. The primary limitation of this study is the moderate sample size of the TNL group, which is to be expected since amniocenteses are not commonly performed in term deliveries. Yet, to overcome such a limitation, we utilized robust differential analysis and functional profiling methods with particular emphasis on controlling the false discovery rate. In addition, our study design (case–control, cross-sectional study) was not ideally suited to discriminate between events that cause labor and those that accompany labor since amniocentesis was performed either in the absence of labor or during labor. Nevertheless, by using robust functional profiling strategies, we provide corroborating evidence for several biological processes previously implicated in the onset of labor, including fetal lung maturity and decidual activation.

Whether the observed association between molecular changes in the amniotic fluid and maternal blood extends to other ethnicities and obstetrical syndromes, such as spontaneous preterm labor and birth, remains to be determined in future studies.

## Conclusion

Collectively, the findings herein provide the first characterization of the amniotic fluid proteome during term parturition, thus demonstrating that such a process is reflected by the altered proteomic composition in this fetal compartment. Moreover, we validated our findings by corroborating the enrichment of specific immune and inflammatory processes associated with labor onset in other intrauterine compartments. Importantly, labor-driven perturbations of the amniotic fluid proteome can be observed in the maternal plasma proteome, thereby supporting its potential use as a biomarker to inform optimal patient management.

## Methods

### Ethics

The study protocol, collection of samples, and use of clinical data were approved by the Human Investigation Committee of Sotero del Rio Hospital, Santiago, Chile (amniotic fluid), and the Institutional Review Boards of Wayne State University and the Pregnancy Research Branch (formerly known as the Perinatology Research Branch), an intramural program of the *Eunice Kennedy Shriver* National Institute of Child Health and Human Development, National Institutes of Health, U.S. Department of Health and Human Services (NICHD/NIH/DHHS), Detroit, MI, USA (maternal plasma). All patients provided written informed consent prior to sample collection. All experiments were performed in accordance with relevant guidelines and regulations.

### Study population

#### Amniotic fluid

Amniotic samples were collected from pregnant women seeking care at the Sotero del Rio Hospital, Santiago, Chile. A cross-sectional study was designed to include 35 women: 11 who underwent amniocentesis at term before the onset of labor and 24 who had an amniocentesis at term after the spontaneous onset of labor (Fig. 1, top panel). Labor was diagnosed by the presence of regular uterine contractions occurring at a frequency of four in 20 min for a minimum of 1 h associated with cervical dilatation and/or effacement changes. Women at term not in labor underwent amniocentesis for the assessment of fetal lung maturity prior to cesarean delivery, whereas those in labor underwent amniocentesis because of uncertain gestational age or for the diagnosis of intra-amniotic infection. Amniotic fluid samples were obtained by transabdominal amniocentesis under ultrasonographic guidance and cultured for the presence of microorganisms (aerobic and anaerobic bacteria and genital Mycoplasmas). Amniotic fluid not used in clinical assessment was centrifuged for 10 min at 4 °C, and the supernatant was aliquoted and stored at − 80 °C until analysis.

We included cases without evidence of medical or obstetrical complications such as preterm labor, preeclampsia, clinical chorioamnionitis, gestational or pregestational diabetes mellitus, positive amniotic fluid culture, meconium-stained amniotic fluid, multiple gestation, and pregnancy with fetal anomalies. The amniotic fluid samples had been previously used in targeted studies of amniotic fluid cytokines and arachidonic acid metabolites^113,115,116,118^.

#### Maternal plasma

Plasma samples were collected from women enrolled in a prospective longitudinal study at the Center for Advanced Obstetrical Care and Research of the NICHD's Pregnancy Research Branch, Detroit Medical Center, and Wayne State University^74^. Only the last sample collected prior to term delivery from women with spontaneous labor (n = 59) and the gestational age-matched samples from women who were not in labor (n = 21) were used (Fig. 1, bottom panel).

The study design is summarized in Fig. 1, which was created with Biorender.com^155^.

### Proteomics

The abundance of 1310 proteins in amniotic fluid samples (75 µL aliquots) and 1125 proteins in maternal plasma samples was determined with the SOMAmer (Slow Off-rate Modified Aptamers) platforms v3 and v2, respectively (SomaLogic, Inc., Boulder, CO, USA), as previously described^44,86^. Briefly, the samples were incubated with SOMAmer mixes pre-immobilized onto streptavidin-coated beads and washed to remove non-specifically bound proteins. Proteins bound to their cognate SOMAmer reagents were tagged by using the NHS-biotin reagent. The beads were treated with an anionic competitor solution to prevent non-specific interactions. The beads were exposed to ultraviolet light to release pure cognate-SOMAmer complexes and unbound SOMAmer reagents, and the photo-cleavage eluate was incubated with a second streptavidin-coated bead to capture the biotinylated proteins. Unbound SOMAmer reagents were removed during subsequent washing, and the bound SOMAmer reagents were separated from their cognate proteins under denaturing conditions and hybridized to custom DNA microarrays. Protein abundance was measured in relative fluorescence units by detecting the Cyanine-3 signal from the fluorophores in SOMAmer reagents. The raw signal intensities were standardized by hybridization control normalization, median signal normalization, and interplate calibration^84^.

### Data analysis

#### Demographic data analysis

Clinical characteristics and demographics of the study participants were summarized as the median and interquartile range for continuous variables and as proportions for categorical variables. To compare data between groups, Welch's t-test and Fisher's exact test were used for continuous and categorical variables, respectively. A p-value < 0.05 was considered statistically significant.

#### Differential protein abundance analysis and validation

The abundance of 1,310 proteins in the amniotic fluid was compared between samples collected from patients at term in labor and from those at term without labor by using moderated t-tests implemented in the *limma* R package^156^. A fold change ≥ 1.25 and a false discovery rate adjusted p-value (q-value) < 0.1 were used to determine statistical significance. The results of differential expression analysis were summarized and visualized with volcano plots and heatmaps, using the R packages *EnhancedVolcano*^157^ and *pheatmap*^158^, respectively.

Amniotic fluid concentrations of four proteins—CXCL10, ELANE, IL-6, and SLPI—were determined previously by specific immunoassays, ELISA, or radioimmunoassay, according to the manufacturer's instructions^113^. These data were used to assess cross-platform reproducibility via Spearman's correlation analysis. The correlations were visualized with scatter plots created with the R package *ggplot2*^159^.

#### Gene Ontology and biological pathway enrichment analysis

All proteins were mapped to Entrez gene identifiers per the manufacturer's provided annotation. We used GSEA^160^ to analyze the molecular signatures database (MSigDB)^161^ C5 sub-collection of gene sets corresponding to GO biological processes, molecular functions, and cellular components. We also performed the same analysis for the MSigDB C2 collection of canonical biological pathways curated from popular online databases, e.g., KEGG and Reactome. The analysis was restricted to gene sets with at least five genes having corresponding proteins measured on the SOMAScan platform. A q-value < 0.25 was considered statistically significant, as recommended by the authors of GSEA^160^. The enrichment of selected biological pathways was visualized as a gene-concept network created using the R/Bioconductor package, *clusterProfiler*^162^.

#### Placental single-cell-specific expression

We previously defined sets of genes as specific to a given population of cells identified by single-cell RNA-seq analyses of the placenta^66^. The log_2_ transformed relative fluorescence units reflecting the abundance of proteins encoded by these genes and measured herein with the SOMAmer assay were standardized by subtracting the mean and dividing by the standard deviation calculated from the reference (TNL) study group^44,79,145^. The standardized values referred to as Z-scores were then averaged and compared between groups with the Wilcoxon rank sum test. A q-value of less than 0.1 was considered significant. To obtain robust summaries of signatures, we considered only those cell types having at least five signature genes with corresponding proteins measured by the SOMAscan platform.

We implemented a previously described strategy^66,69^ to define an amniotic fluid labor signature consisting of the top 20 proteins most increased in abundance after the onset of labor. Of these 20 amniotic fluid proteins, 15 (HNRNPA2B1, MMP8, SFTPD, PRTN3, CSF3, PEBP1, ACY1, PPIA, TPT1, CTSV, ICOSLG, GDI2, CXCL8, MMP1, and AKR1A1) were also measured in maternal plasma by using the SOMAScan platform (v2) (Fig. 1)^74^. The average z-scores corresponding to these 15 proteins were then compared between the TIL and TNL groups with Wilcoxon tests and visualized in boxplots created with the R package, *ggpubr *^163^.

#### Plasma proteomic models to classify labor and no labor groups

A random forest model was fit to discriminate between the labor and no labor groups by using protein data derived from the plasma samples. Only amniotic fluid proteins that significantly increased in abundance with labor were considered because there was no significant difference between the labor groups in the aggregated Z-score in the maternal plasma proteome of the 20 amniotic fluid proteins that decreased in abundance with labor. A LOOCV procedure was used to assess the model's generalizability to unseen data. Briefly, in each iteration of LOOCV, one patient profile was excluded from the training set used to fit the model, and the resulting model was applied to the data of the patient excluded from the training set. During training, an initial random forest classifier was fit, and the top 10 features, ranked according to metric importance, were selected to fit the final model. The procedure was repeated for all patients, and the model performance was evaluated by calculating the area under the receiver operating curve (AUROC). The receiver operating curve was plotted using the R package, *pROC*^164^, and the R packages, *randomForest*^165^*,* and *ranger*^166^, were used to fit the random forest models.

## Supplementary Information

Supplementary Information 1.

Supplementary Information 2.

Supplementary Information 3.

Supplementary Information 4.

Supplementary Information 5.

Supplementary Information 6.

Supplementary Information 7.

Supplementary Information 8.

Supplementary Information 9.

Supplementary Information 10.

## Data Availability

The amniotic fluid and the maternal plasma proteomics data presented in this study has been provided as Supplementary Tables S6 and S7.
