# Supplementary figures and images for: The amniotic fluid proteome changes with term labor and informs biomarker discovery in maternal plasma
Source: Sci Rep. 2023 Feb 23;13:3136. doi: 10.1038/s41598-023-28157-3 (PMC9950459; doi:10.1038/s41598-023-28157-3)

Principal component analysis

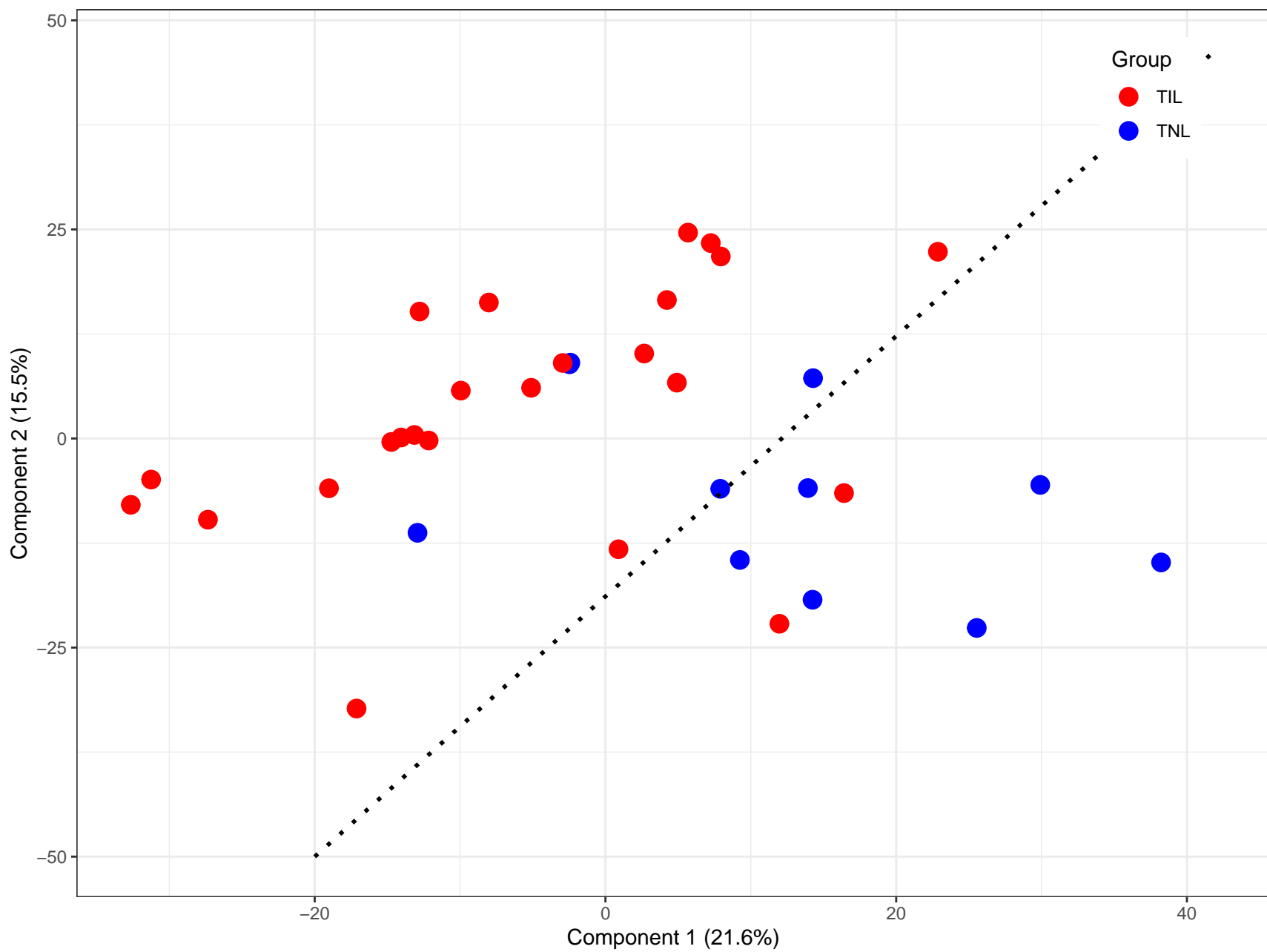

Supplement: Supplementary file 1 — Supplementary Information 1. [file 41598_2023_28157_MOESM1_ESM.pdf]
