# Supplementary material for: The amniotic fluid proteome changes with term labor and informs biomarker discovery in maternal plasma
Source: Sci Rep. 2023 Feb 23;13:3136. doi: 10.1038/s41598-023-28157-3 (PMC9950459; doi:10.1038/s41598-023-28157-3)
Supplement: Supplementary file 9 — Supplementary Information 9. [file 41598_2023_28157_MOESM9_ESM.doc]

**Legend**

**Supplementary Figure 1:** The amniotic fluid proteome distinguishes term labor from term without labor. Amniotic fluid proteomes from term without labor (TNL, blue dots) and term labor (TIL, red dots) patients are depicted as their first and second principal components. The proportion of variance explained by each principal component is shown along the axis. The dotted line shows the logistic regression decision boundary for separating TIL and TNL groups.
