# Supplementary material for: The amniotic fluid proteome changes with term labor and informs biomarker discovery in maternal plasma
Source: Sci Rep. 2023 Feb 23;13:3136. doi: 10.1038/s41598-023-28157-3 (PMC9950459; doi:10.1038/s41598-023-28157-3)
Supplement: Supplementary file 10 — Supplementary Information 10. [file 41598_2023_28157_MOESM10_ESM.doc]

**Supplementary Table Legends**

**Supplementary Table 1. Demographic characteristics of the maternal plasma study cohort.** Continuous variables were compared using Welch’s t-test and are summarized as medians (interquartile range). Categorical variables are shown as number (%) and were compared using Fisher’s exact test.

**Supplementary Table 2: Proteins significantly changing in abundance between** **amniotic fluid samples collected at term in labor (TIL) and term not in labor (TNL).** The table includes the manufacturer identifier (ID), gene symbol, protein name, ENTREZ database identifier, log_2_ fold change, nominal (p), and adjusted p-value (q-value).

**Supplementary Table 3:** **Gene ontology enrichment analysis of labor-associated changes in the amniotic fluid proteome.** Description: name of the gene ontology term; SIZE: number of genes belonging to the gene ontology after excluding genes whose corresponding proteins were not assayed. NES: normalized enrichment score that represents the degree to which the gene ontology is overrepresented at the top or bottom of the proteins ranked according to the t-statistic. qvalues: False discovery rate adjusted nominal significance of the enrichment score. core_enrichment: a list of the proteins that contributed the most to the enrichment of the gene ontology.

**Supplementary Table 4: Biological pathway analysis of labor-associated changes in the amniotic fluid proteome.** Description: name of the biological pathway; SIZE: number of genes belonging to the biological pathway after excluding genes whose corresponding proteins were not assayed. NES: normalized enrichment score representing the degree to which the biological pathway is overrepresented at the top or bottom of the proteins ranked according to the t-statistic. qvalues: False discovery rate adjusted nominal significance of the enrichment score. core_enrichment: a list of the proteins that contributed the most to the enrichment of the biological pathway.

**Supplementary Table 5: Labor-associated changes in the meta-protein abundance of RNA Seq single-cell signatures.** The table includes the cell type, symbols of marker genes, nominal p-values (Wilcoxon rank sum test), false discovery rate adjusted p-values (q-value), and the direction of change.

**Supplementary Table 6: Amniotic fluid proteomics data presented in this study**. The table includes the log_2_ transformed protein abundance data for each sample (rows) and each of the 1310 proteins. ID: anonymized identifier indicator of the pregnant woman, Group: term in labor (TIL) and term not in labor (TNL).

**Supplementary Table 7: Maternal plasma proteomics data presented in this study**. The table includes the log_2_ transformed protein abundance data for each sample (rows) and 81 proteins identified as significantly increased with labor in the amniotic fluid. ID: anonymized identifier indicator of the pregnant woman, Group: term in labor (TIL) and term not in labor (TNL).
